# Comparative Study of Hydrolysis-Based Methods Coupled with QuEChERS Extraction Followed by GC–MS/MS and LC–MS/MS for the Determination of Complex Pesticide Residues in Melons and Dry Beans

**DOI:** 10.3390/foods15081314

**Published:** 2026-04-10

**Authors:** Iwona Wenio, Daria Dawidziak, Dorota Derewiaka, Ewa Majewska, Iwona Bartosiewicz

**Affiliations:** 1 Department of Technology and Food Assessment, Faculty of Food Technology, Institute of Food Sciences, University of Life Science, 02-787 Warsaw, Poland; dorota_derewiaka@sggw.edu.pl (D.D.); ewa_majewska1@sggw.edu.pl (E.M.); 2 Voivodeship Sanitary and Epidemiological Station, 00-875 Warsaw, Poland; daria.dawidziak@sanepid.gov.pl (D.D.); iwona.bartosiewicz@sanepid.gov.pl (I.B.)

**Keywords:** pesticide residue, acidic, alkaline, enzymatic hydrolysis, LC-MS/MS, GC-MS/MS, food

## Abstract

Determination of complex pesticide residues in food matrices poses a considerable analytical challenge, primarily because the analytes exhibit diverse physicochemical properties. Monitoring pesticides across a wide range is essential to meet all regulatory requirements and safeguard consumer health. One of the most promising analytical approaches is the hydrolysis of compounds, particularly acidic hydrolysis, which enables the identification of a broad range of substances that pose significant analytical challenges. In addition, pesticide residues may interact with food matrix components, leading to the formation of conjugated forms such as ester- or glycoside-bound compounds. Therefore, the development of appropriate analytical strategies, including hydrolytic steps, is essential to release these bound residues and enable the determination of complex residue definitions comprising multiple related compounds. Furthermore, this study compares different hydrolysis strategies, including enzymatic, acidic, and alkaline hydrolysis, in order to assess their suitability for the determination of complex pesticide residue definitions using a QuEChERS-based (Quick, Easy, Cheap, Effective, Rugged, Safe) extraction approach. The given methodology meets all criteria listed in the Document SANTE 11312/2021 v2026. The procedure allows for good measurement precision relative standard deviation (RSD < 20%), and recovery in the scope ranging from 55.6% to 107.8% acidic hydrolysis and 51.7% to 100.8% for alkaline hydrolysis and 35.1–108.9% for enzymatic hydrolysis depending on the experimental variant, and limit of quantification (LOQ) as low as 10 to 100 µg/kg for the determination of ten complex definitions of pesticides with the use of liquid chromatography mass spectrometry (LC-MS/MS) and gas chromatography mass spectrometry (GC-MS/MS) analytical methods.

## 1. Introduction

Pesticides are among the most commonly used chemicals in the home and garden sectors, with herbicides being particularly prevalent. Among these, 2,4-D is particularly important, as it is one of the most widely used herbicides worldwide and is commonly applied in non-agricultural environments, including urban landscapes such as lawns, home gardens, and recreational areas [1]. All the above-mentioned herbicides act by interfering with critical metabolic pathways in plants, effectively controlling unwanted vegetation in both domestic and commercial settings. Specifically, 2,4-D belongs to a broad class of acidic herbicides—compounds typically defined by the presence of functional groups such as free acids, esters, salts, or conjugated derivatives. This structural diversity affects their solubility, environmental fate, and biological activity. 2,4-D, as a representative of this group, undergoes biotransformation in both plants and soil, leading to the formation of various conjugates and metabolites. Common conjugates include glucose and amino acid derivatives, formed by natural detoxification processes in plants to reduce the compounds’ phytotoxicity. Additionally, 2,4-D can be metabolized into compounds such as 2,4-dichlorophenol (2,4-DCP), which is a key environmental degradation product. The formation of such metabolites and conjugates not only influences the herbicide’s persistence and mobility in soil and water but also has implications for environmental safety and regulatory assessment. This complexity in metabolism and chemical forms underscores the importance of considering both parent compounds and their derivatives when evaluating the behavior and impact of acidic pesticides such as 2,4-D in non-agricultural applications [2,3].

The maximum permissible pesticide residue levels (MRLs) in European Union legislation are established in Regulation (EC) No 396/2005 of the European Parliament and of the Council [4]. However, due to the continuously expanding range of pesticide residues and active substances, the scope of this Regulation, despite ongoing updates, remains incomplete and is subject to continual revision. The European Food Safety Authority (EFSA) determines the maximum permissible pesticide residue levels through chronic and acute risk assessments (short-term and long-term consumption) using the EFSA PRIMo revision 3 model [5]. A practical, selective analytical method must be employed to assess the risk of a particular compound, enabling precise detection of its content with minimal measurement uncertainty, according to OECD Guideline Test N° 507 [6] and SANTE/11312/2021 v2026 guidelines, which were announced to formalize the rules for the implementation of analytical methods [7]. It is known that many pesticide residue compounds specifically interact with food matrices, forming distinct chemical compounds [8]. Through interactions with pesticide residues, animal food matrices form various conjugates as metabolic pathways within cells and bodily fluids are altered. These transformations result in the formation of compounds containing glycosidic groups, amino acids, and peptides. When analyzing plant matrices, the largest group of transformed compounds consists of esters and glycosides [9]. An appropriate analytical method must be used to correctly determine these complex compounds, implementing one of the analytical variants that directly measures all the compounds of interest [10].

A solution may lie in a hydrolysis-based approach, which provides a scientifically sound means of releasing analytes from conjugated, esterified, or amidated forms, thus enabling reliable identification and quantification in accordance with comprehensive residue definitions. To confirm that a given analytical method meets established criteria, it is necessary to demonstrate that each component undergoes efficient extraction and hydrolysis. Hydrolysis may be required when esters, amides, or conjugates are included in the residue definition, mainly if their chemical structures are unknown or analytical standards are unavailable. Available reference standards can be used for direct quantification or comparative analysis before and after hydrolysis to verify hydrolysis efficiency. If standards are unavailable but identifiable, hydrolytic conditions are used in metabolism studies, and efficiency can be assumed if previously characterized, key metabolites are available. If conditions differ from those in metabolism studies, a cross-validation study must be conducted using incurred residues and comparing hydrolytic conditions to extreme scenarios, such as strong acids, alkaline or enzymatic hydrolysis [11]. Conditions are considered sufficient if no additional cleavage of conjugates or esters occurs with an acceptable deviation of ±20% difference in recovery. These procedures ensure analytical reliability and compliance with regulatory requirements for residue assessment in agricultural and environmental matrices (European Commission SANCO, 2023) [12].

Research on pesticide metabolism in plant leaves has shown that ester variants are similar, with the parent ester hydrolyzing into its corresponding acid [13]. The transformation and fate of these compounds can be traced using carbon-14 labeling, allowing precise determination of ester ratios in plant extracts [14]. Additionally, studies are being conducted to assess the stability of conjugates and esters in food and their degradation during processing and thermal treatment [15].

To improve risk assessment, it is essential to address data gaps concerning the toxicological significance of metabolites. The MRL review indicates that, since conjugates primarily degrade into the acidic form, storage stability studies should include all compounds classified as residues, including conjugates [16].

Chlorophenoxy herbicides such as 2,4-D, MCPA, dichlorprop, and related compounds are generally characterized by low acute toxicity. Experimental studies demonstrate that 2,4-D induces oxidative stress and hepatotoxicity, including disruption of antioxidant systems and lipid metabolism [17,18]. At the cellular level, both 2,4-D and MCPA act as uncouplers of oxidative phosphorylation, indicating mitochondrial dysfunction even at low concentrations [19]. Epidemiological and toxicological reviews suggest potential associations between exposure to phenoxy herbicides and increased risks of certain cancers (e.g., soft-tissue sarcoma, Hodgkin’s disease, lymphoma) and adverse reproductive outcomes, although causal relationships remain inconclusive. Additionally, biomonitoring studies indicate that these compounds are readily absorbed and metabolized, potentially influencing their systemic effects and toxicokinetics [20].

According to EFSA data from 2022–2023, the residue levels of the analyzed pesticides in food crops were generally below the established MRLs, although occasional exceedances were reported for 2,4-D, 2-phenylophenol, dichlorprop, MCPA and MCPB, haloxyfop, quizalofop, and fluoxypyr. These findings are summarized in the EFSA report table “Pesticide Residue Exceedances” (EFSA, 2022, 2023), which provides the number of samples tested, the number of detected residues, and the incidence of exceedances (Table 1). This information highlights potential dietary exposure and supports risk assessment for consumers, emphasizing the need for continuous monitoring of pesticide residues in food matrices [21,22].

Pesticide residues in plants undergo the following metabolic transformations: canonical Phase I metabolism, Phase II conjugation, Phase III transport, and non-canonical Phase IV. The epigenetic mechanisms underlying these transformations are a potential area of study [23]. In Phase I reactions, cytochrome P450 monooxygenases catalyse redox reactions, converting hydrophobic chemicals into less hydrophobic metabolites through hydroxylation. Phase II metabolism involves conjugation with thiols. In higher plants, most pesticides bind to thiols such as glutathione (GSH) and cysteine (Cys); however, their affinity for thiols varies depending on the type of pesticide [24]. The interaction between pesticide molecules and glutathione is catalysed explicitly by the glutathione S-transferase (GST) subfamily of proteins [25]. Glycosyltransferases catalyse the conjugation of sugars with endogenous metabolites or exogenous chemicals in plants. The function of glycosylation is to increase the solubility of substrates in water, facilitating their metabolism [26]. Condensation is another type of Phase II reaction that links amino/carboxyl groups to xenobiotics to detoxify pesticides such as serine, alanine, aspartic acid, glutamate, threonine, and tryptophan, forming conjugates. Acetylation is an essential metabolic process for the metabolism of pesticides or their metabolites containing primary amine groups, such as fatty or aromatic amines, sulphonamides, hydrazines, acylhydrazines, or amino acids, making the pesticide less soluble in water [27]. Methylation is a biochemical modification catalysed by methyltransferases using S-adenosyl-L-methionine (SAM) as a methyl donor. From a physicochemical perspective, methylation weakens the polarity of the precursor metabolite, thereby reducing its excretion or intracellular mobility [28].

In this study, acidic, alkaline, and enzymatic hydrolysis strategies were comparatively evaluated for the determination of complex pesticide residues using QuEChERS extraction coupled with LC-MS/MS and GC-MS/MS detection. The comparison focused on analytical approaches enabling the simultaneous determination of acidic pesticides and their conjugated forms after an initial hydrolysis step followed by QuEChERS extraction. In particular, the alkaline hydrolysis procedure described by Steinborn et al. (2017) was considered as a reference approach, as it efficiently converts ester and conjugated residues into their corresponding free acids prior to analysis. Under optimized conditions (approximately 30 min at 40 °C), high conversion efficiencies (>90%) were reported, resulting in significantly improved quantification of residue levels compared with conventional QuEChERS methods without hydrolysis [29].

Enzymatic deconjugation using specific β-glucosidase mixtures has been proposed as a milder alternative to alkaline hydrolysis, enabling the release of parent herbicides from glucoside conjugates while minimizing degradation of labile functional groups [30]. Their study demonstrated that enzymatic treatment can efficiently cleave glucoside conjugates and improve the detection of bound residues, while maintaining good analytical performance in accordance with SANTE validation criteria. In the context of the present study, this approach was considered alongside acidic and alkaline hydrolysis in order to evaluate its suitability for the comprehensive determination of pesticide residues and their conjugated forms in complex matrices using QuEChERS extraction coupled with LC-MS/MS and GC-MS/MS detection [30].

Hydrolysis can be conducted using acid, alkaline, or a combination of methods, under both conditions. When selecting hydrolytic conditions, it is crucial to optimize parameters such as extraction time, temperature, solvent type, component concentrations, and food matrix type. Because bond degradation is necessary, hydrolysis conditions must be relatively stringent.

The aim of the study was to select the optimal extraction method and hydrolysis type for pesticides in plant material, particularly melons and dry beans. The study undertaken explored the selection of the optimal hydrolytic extraction method to achieve the highest recoveries.

## 2. Materials and Methods

### 2.1. Research Material

The analytical material, consisting of melons and dry beans, each at 500 g, was purchased from a local organic store and analysed for contamination with the pesticides under investigation. The edible parts of the melon and dry beans were homogenized using a grinder with dry ice in a 2:1 (*v*/*v*) ratio, then placed in polyethylene containers and stored at −18 °C. The preparation of the analytical sample was provided by the Codex document CAC/GL 33 [31,32]. The melon matrix was used in the preliminary experiment, in which five samples were subjected to enzymatic hydrolysis. Dry beans were used in the main experiment and were subjected to both acid and alkaline hydrolysis.

### 2.2. Sample Pretreatment

As part of the assessment of optimal performance for pesticide residue detection with complex definitions presented in Table 2, three alternative analytical methods were employed. Samples were prepared using enzymatic, alkaline, and acid hydrolysis. Due to the limited stability and short-term activity of the enzymes (α-amylase, α-galactosidase, and β-glucosidase), a full validation of the enzymatic hydrolysis procedure was not possible. Instead, the method performance was preliminarily assessed by conducting five replicate determinations at the LOQ level, and the mean value was reported.

### 2.3. Reagents and Materials

High-purity (96.0–99.9%) standards for 42 pesticides were purchased from Dr. Ehrenstorfer (LGC Standards, Warsaw, Poland). The selection of the 42 analytes was based on regulatory relevance and commercial availability. Only pesticides that are commercially available and included in residue definitions established under EU legislation were considered. The selection followed the requirements of Regulation (EC) No 396/2005 and the analytical guidance provided in the European Commission SANTE document (SANTE/11312/2021 v2026), which recommends determining parent compounds together with relevant metabolites, esters, and conjugated forms included in official residue definitions. Acetonitrile and water (LC-grade, purity ≥ 99.9%) were purchased from JT Baker Chemicals. QuEChERs salts 1st and 2nd (4 g magnesium sulphate, 1 g sodium chloride, 1 g trisodium citrate dihydrate, 0.5 g disodium hydrogen citrate, and 6 g sodium sulphate, 0.9 g magnesium sulphate, 0.15 g PSA), sodium hydroxide, concentrated acetic acid and sulfuric acid (VI), (purity 99.9%), were purchased from Merck, Warsaw, Poland. β-Glucosidase (from almonds), β-galactosidase (from Aspergillus oryzae), and α-amylase (from Aspergillus oryzae) were purchased from Merck Life Science (Warsaw, Poland) and used without further purification. The extracts were filtered through 0.22 μm PTFE syringe filters (Millex-LG, hydrophilic PTFE membrane, polypropylene housing; Merck Millipore, purchased from Merck Life Science, Warsaw, Poland). A multi-analyte stock and working solutions were prepared in acetonitrile and stored at 4 °C until use (Table 2). A multi-analyte stock (Table 2) and working solutions for calibration standard (Table 3) were prepared in acetonitrile and stored at 4 °C until use.

#### 2.3.1. Acidic QuEChERS Extraction

An analytical sample (2.0 ± 0.01 g) was transferred into a 50 mL polypropylene test tube. Spiking standards corresponding to the concentrations in Table 3 were added for recovery measurements. Spiking was performed at three levels: LOQ, 10 × LOQ, and 100 × LOQ. Samples with high protein content (beans) were hydrated with 20 mL of LC-grade water. Each sample was then mixed with 10 mL 1% (*v*/*v*) acetic acid in acetonitrile and 2 mL of 5 M sulfuric acid. Test tubes were then placed in a water bath with shaking for 16 h at 60 °C. After the incubation, 1st QuEChERs salts (1 g magnesium sulphate, 1 g sodium chloride, 1 g trisodium citrate dihydrate, 0.5 g disodium hydrogen citrate, and 6 g sodium sulphate) were added. The samples were then cooled in an ice-water bath, shaken for 1 min, and centrifuged for 5 min at 10 °C and 4000 rpm. A 6 mL aliquot of the supernatant was then transferred to a 15 mL polypropylene tube containing 2nd QuEChERs salts (900 mg magnesium sulphate, 150 mg PSA), shaken for 1 min, and centrifuged for 5 min at 10 °C at 4000 rpm. After purification, samples were cooled down in an ice-water bath and then pH-adjusted. Samples of pH < 1 were neutralized by the addition of 2 mL of 5 M sodium hydroxide. Nevertheless, the presence of PSA in the 2nd round of QuEChERs purification was sufficient to yield a pH of approximately 4.5 for the majority of food matrices. The samples were then frozen for 1 h at −20 °C, followed by 1 min shaking, 5 min centrifugation at 10 °C at 4000 rpm, and filtration of the final extract through a 0.2 µm PTFE filter. Such prepared extracts were then analysed using liquid chromatography coupled with tandem mass spectrometry (LC-MS/MS) and gas chromatography coupled with tandem mass spectrometry (GC-MS/MS). The sample preparation procedure was developed in-house.

#### 2.3.2. Alkaline QuEChERS Extraction

Alkaline QuEChERS extraction was employed to isolate acidic pesticides from various matrices, including fruits and cereal-based products such as melons, dry beans, broad beans, and canola oil. Prior to extraction, all samples were homogenized and stored at 4 °C. For the analysis, 5.00 ± 0.05 g of dry matrices (e.g., cereals or flour) was accurately weighed into a 50 mL polypropylene centrifuge tube. To dry samples, 10.0 mL of deionized water was added, followed by thorough homogenization and a 15 min equilibration at room temperature. For spiked samples corresponding to the concentrations in Table 3, analytical standards were added at this stage, followed by gentle mixing and a 30 min stabilization period. Subsequently, 10.0 mL of acetonitrile was added to each tube. The pH was adjusted to 12.0–12.5 using a 5 M sodium hydroxide solution. Tubes were sealed, briefly vortexed, and shaken on a mechanical shaker for 1 min. Alkaline hydrolysis was then performed by incubating the tubes in a shaking water bath at 60 °C for 1 h at 120–180 rpm. After hydrolysis, samples were cooled to room temperature and acidified with 5 M sulfuric acid to a pH of approximately 2. The tubes were then vortexed for 1 min, and the QuEChERS extraction procedure was performed by adding a salt mixture containing 4 g anhydrous MgSO_4_, 1 g NaCl, 1 g trisodium citrate dihydrate (TSCD), and 0.5 g disodium hydrogen citrate sesquihydrate (DHS). The samples were cooled in an ice bath for 5 min, shaken for 2 min, and centrifuged at 4000 rpm for 5 min at 10 °C. Subsequently, 6 mL of the organic extract was transferred to a clean tube containing dispersive solid-phase extraction sorbents (900 mg MgSO_4_ and 150 mg PSA) for clean-up. The mixture was again cooled in an ice bath for 5 min, shaken for 2 min, and centrifuged under the same conditions. Finally, the purified extract was filtered through a 0.22 µm PTFE syringe filter and analyzed by LC-MS/MS The extraction conditions were optimized and modified according to the procedure reported by Steinborn et al. (2017) [29].

#### 2.3.3. Enzymatic QuEChERS Extraction

The enzymatic extraction procedure was evaluated using four experimental variants that differed in the application of enzymatic and chemical hydrolysis. Initially, 10 mL of acetate buffer (pH 4) was added to the sample to provide suitable conditions for enzymatic activity. For spiked samples, analytical standards were added at this stage to achieve the target fortification levels, then the samples were gently mixed. In Option 1, enzymatic hydrolysis was performed by adding a mixture of β-glucosidase, β-galactosidase, and α-amylase, each at 5 U mL^−1^. The samples were incubated for 4 h at 37 °C in a shaking water bath to facilitate enzymatic cleavage of conjugated compounds. In Options 2–4, no enzymes were added. Following this step, extraction was initiated by adding 10 mL of 1% acetic acid in acetonitrile. Depending on the variant, either 2 mL of deionized water (Options 1 and 4) or 2 mL of 5 M sulfuric acid (Options 2 and 3) was introduced to promote different hydrolysis conditions (Table 4). The mixtures were subsequently incubated for 16 h at 60 °C in a shaking water bath to ensure efficient release of analytes from the sample matrix. After hydrolysis, purification was performed using the QuEChERS extraction method, as described in Section 2.3.2, and the samples were subsequently analyzed by LC–MS/MS. The extraction conditions were optimized and modified according to the procedure reported by Aloisi et al. (2024) [30].

### 2.4. LC-MS/MS Analysis

Chromatographic separation and quantification of the target pesticide residues were performed using a liquid chromatography system (Agilent 1260 Infinity II, Saint Louis, MO, USA) equipped with a G7112B binary pump, G7129A autosampler, and G7116A thermostated column compartment. The LC system was coupled to an Agilent 6470B triple quadrupole mass spectrometer (QqQ) with an electrospray ionization (ESI) source operating in both positive and negative ionization modes. Chromatographic separation was achieved using an Agilent Zorbax Eclipse Plus C18 column (150 mm × 2.1 mm, 1.8 μm particle size) protected by a pre-column 1290 Infinity II online filter (2 mm ID × 0.3 μm dp). The column was maintained at 55 °C. The mobile phase consisted of water with 5 mM ammonium formate and 0.1% formic acid (phase A) and methanol (phase B). The elution was carried out at a flow rate of 0.2 mL min^−1^ using the following gradient: 5% phase B held for 0.1 min, increased to 90% phase B over 16 min, held at 90% phase B for 3 min, followed by 6 min equilibration at 5% phase B, with a total run time of 25 min. The injection volume was 2 μL. The ESI source parameters were optimized as follows: drying gas temperature 250 °C, sheath gas temperature 300 °C, heat block temperature 400 °C, drying gas flow (N_2_) 6 L min^−1^, sheath gas flow (N_2_) 11 L min^−1^, and auxiliary heating gas flow (dry air) 10 L min^−1^. The capillary voltage was set to +3000 V in positive-ion mode and −3500 V in negative-ion mode. Nozzle voltage was maintained at 500 V, and delta EMV was set to ±200 V, depending on polarity. The mass spectrometer operated in multiple reaction monitoring (MRM) mode with two transitions (quantifier and qualifier ions) monitored per compound. The acquisition frequency was set to 0.55 Hz with an optimized dwell time of at least 20 ms per transition. Retention times, MRM transitions, and collision energies for all analytes are detailed in Table 5. Data acquisition, instrument control, and quantification were performed using Agilent MassHunter Workstation Software (version 12). Samples were stored and processed at 10 °C for 24 h, during which no significant degradation of analytes was observed. No carryover was detected in solvent blanks run after high-concentration samples. The system was calibrated daily, and quality control samples were included in each batch to monitor instrument performance. Table 6 presents the operational parameters of the liquid chromatograph–tandem mass spectrometer (LC-MS/MS), highlighting the most intense multiple reaction monitoring (MRM) transitions and their corresponding collision energies. For each target analyte, the following data are provided: retention time (RT, min.), ionization polarity, selected reaction monitoring transitions (SRMs), fragmentor voltage, collision cell accelerator voltage (CAV, V), and collision energy (CE, eV). Every analyte in Table 3 was determined using LC–MS/MS, except for 2-phenylphenol, which was analyzed by GC–MS/MS (Table 5).

### 2.5. GC-MS/MS Analysis

Gas chromatographic separation and quantification were performed using a Shimadzu Nexis GC-2030 (Shimadzu, Kyoto, Japan) system equipped with an AOC-6000 Plus autosampler and coupled to a GCMS-TQ8040NX triple quadrupole mass spectrometer (QqQ). The mass spectrometer operated in tandem mass spectrometry (MS/MS) mode with electron ionization (EI) for compound detection and quantification. The injector temperature was set at 250 °C. Splitless mode injection for 1 min, was used with an injection volume of 1 μL. Separation was performed on an SH-I-5MS (Shimadzu Corporation, Kyoto, Japan) fused silica capillary column (30 m × 0.25 mm) with a 0.25 µm film thickness (Shimadzu). Helium (purity 99.9995%) was used as the carrier gas at a constant-pressure flow rate of 1.4 mL/min. The column oven temperature program was as follows: the initial temperature was set to 105 °C and maintained for 3 min; then, it was increased to 130 °C at 10 °C/min, increased to 200 °C at a rate of 4 °C/min, increased to 280 °C at a rate of 8 °C/min, and held for 10 min. The total program time was 43 min. The mass spectrometer was operated with an electron impact (EI) source using the multiple reaction monitoring (MRM) mode. Table 2 presents the characterization of the ions used for qualitative analysis of the investigated pesticides. The ion source and transfer line temperatures were set at 225 °C and 280 °C, respectively. Each target compound was monitored in the multiple reaction monitoring (MRM) mode for quantification and identification. LabSolutions GC-MS (version 4.20) was used for instrument control, data acquisition, and processing. The identification of target compounds relied on comparing the retention times (tR) of chromatographic peaks to those of reference standards to estimate and qualify ions. Each standard was injected twice at the beginning and end of the validation sequence. Table 3 presents the operational parameters of the tandem mass spectrometer GC-MS/MS, specifically highlighting the most intense transitions in multiple reaction monitoring (MRM) and the corresponding collision energies. The Kovats retention index (IR) and retention time (tR) are also provided for each analysed compound. GC–MS/MS analysis was applied only for 2-phenylphenol due to its thermal stability and suitability for gas chromatographic separation.

## 3. Validation Parameters

The developed LC-QqQ-MS/MS method was validated in accordance with the European Commission’s SANTE/11312/2021 v2026 guidelines (European Commission, 2026) for the determination of pesticide residues in food matrices. The validation covered key performance parameters, including selectivity, linearity, limits of detection (LOD) and quantification (LOQ), precision, accuracy, extraction recovery, matrix effects, and stability. Selectivity was assessed by analyzing 20 blank matrix samples from various fruit and vegetable types. Additionally, reagent samples were analyzed to confirm the purity of the reagents used. No endogenous interferences were observed at the analyte retention times. All signals were below 30% of the response at the LOQ level, confirming the method’s specificity and absence of matrix interferences. Control samples were included in each analyzed batch to ensure method reliability. These consisted of blanks, spiked samples (blank matrix fortified with known analyte concentrations), and replicates. Blank samples showed no detectable signal, confirming absence of contamination.

Linearity was evaluated by constructing matrix-matched calibration curves at five concentration levels ranging from LOQ to 10 × LOQ. Calibration was performed using weighted (1/×) linear regression. All analytes demonstrated excellent linearity, with correlation coefficients (R^2^) exceeding 0.99. To prepare the calibration curve, a different composition of spiking standards (Table 3) was used to ensure that the total extraction efficiency of conjugated esters and metabolites was 100%. Linearity was further confirmed across a broader dynamic range of 10–1000 ng/kg for selected compounds, with consistent performance across matrices. LOD and LOQ were determined based on signal-to-noise criteria and method performance. The LOD was defined as the lowest concentration with an S/N ratio greater than 3, while the LOQ corresponded to the lowest validated level showing acceptable precision (coefficient of variation, CV < 20%) and accuracy (bias within ±20%). The LOQ values ranged from 10 to 100 µg/kg, depending on the compound (Table 3). Precision was evaluated in terms of repeatability (intra-day). Repeatability was assessed by analyzing five replicate samples spiked at three levels (LOQ, 10 × LOQ, and 100 × LOQ) on a single day. Intermediate precision was assessed across three different days. In all cases, the relative standard deviation (RSD) remained below 20%, fulfilling the SANTE (European Commission SANTE, 2026) criteria. Accuracy was assessed through recovery experiments at the same three spiking levels using five replicates per level. Mean recoveries for many of the analysed compounds ranged from 70% to 120%, confirming the method’s accuracy. Extraction recovery was determined by comparing the analyte responses in spiked matrix extracts before and after extraction. The recoveries were consistent and reproducible across different matrices and spiking levels. Matrix effects (ME%) were assessed by comparing the slopes of calibration curves prepared in matrix extracts (matrix-matched standards) with those obtained from standards prepared in acetonitrile (pure solvent). Most analytes showed matrix effects within ±20%, indicating acceptable ion suppression or enhancement. To compensate for matrix-induced signal variations, quantification was performed using matrix-matched calibration prepared in blank matrix extracts processed according to the same sample preparation procedure as the analytical samples. Where necessary, matrix-matched calibration was employed to compensate for these effects. Calibration standards were prepared in blank matrix extracts, using the same sample preparation procedure as for the analytical samples. Short-term stability of extracts was confirmed for at least 24 h at 4 °C and during autosampler storage at 10 °C. Long-term stability of stock solutions and fortified samples was also evaluated during method development, and the variability did not exceed the acceptable limit. The validation results demonstrated that the analytical method is selective, sensitive, accurate, and precise, and suitable for routine pesticide residue quantification in complex food matrices.

## 4. Results and Discussion

### 4.1. Comparison of Results Obtained in Different Hydrolysis Approaches

The analytical performance of the developed LC-QqQ-MS/MS method was assessed through a comparative validation of two modified QuEChERS extraction protocols: acidified and alkaline. The experimental data, as presented in Table 4 and Table 5, reveal pronounced differences in extraction efficiency, precision, and detection sensitivity between the two approaches, with the alkaline protocol providing a distinct analytical advantage for several classes of acidic herbicides. A comparative evaluation of acid and alkaline hydrolysis protocols for the determination of acidic pesticide residues in legume matrices reveals clear analytical advantages of the acid-based approach, including greater precision and clearer signals. Although both methods yield acceptable recoveries (REC 30–140% and RSD ≤ 20%), the acidic hydrolysis method consistently demonstrated superior repeatability and chromatographic performance. Quantitative recoveries for most analytes were within acceptable ranges for both hydrolysis conditions. Specifically, acidic hydrolysis yielded average recoveries of 57.5% for quizalofop, 104.3% for haloxyfop, 90.4% for 2,4-D, 70.2% for MCPA, 78.7% for MCPB, and 94.0% for bentazon. In comparison, average recoveries obtained via alkaline hydrolysis were slightly higher: 78.7% for quizalofop, 87.7% for MCPB, and slightly lower for 71.5% for 2,4-D, 86.6% for haloxyfop, 65.5% for MCPA, and 93.7% for bentazon. More critically, the acid hydrolysis protocol exhibited substantially improved precision across all analytes, as evidenced by lower relative standard deviations (RSD). The average RSD values for quizalofop, haloxyfop, 2,4-D, MCPA, MCPB, and bentazon under acidic hydrolysis were 3.3%, 5.4%, 1.8%, 1.0%, 2.8%, and 2.1%, respectively. In contrast, the corresponding average RSDs for the alkaline hydrolysis approach were markedly higher: 8.5% for quizalofop, 11.1% for haloxyfop, 4.8% for 2,4-D, 6.2% for MCPA, 2.7% for MCPB, and 2.2% for bentazon. These results suggest that the alkaline method is more variable, likely due to inconsistent matrix effects and less controlled analyte stability under strongly basic conditions. Extracts subjected to acid hydrolysis consistently yielded sharp, symmetrical peaks with minimal baseline noise, indicating efficient purification and minimal matrix interference (Table 7). Conversely, chromatograms derived from alkaline hydrolysis displayed broader peaks, elevated baselines, and in some cases, retention time shifts—all indicative of greater co-extraction of interfering substances. Although alkaline hydrolysis may offer slightly higher recoveries for certain compounds, particularly those existing in conjugated forms, this benefit is counterbalanced by reduced repeatability and increased susceptibility to matrix effects. In contrast, acidic hydrolysis achieves a favourable balance between sufficient analyte release, low RSD values (<3%), and superior chromatographic performance, making it the preferred approach for routine monitoring and regulatory compliance in complex plant-derived matrices. The LODs and LOQs derived from both extraction strategies were within or below the maximum residue limits (MRLs) set by EU regulations, confirming the method’s suitability for regulatory applications.

Aloisi and Mol (2024) proposed enzymatic deconjugation as a mild alternative to conventional alkaline hydrolysis for the determination of glucosidic metabolites of acidic herbicides [30]. Specifically, a mixture of α- and β-glucosidases derived from *Aspergillus niger* was applied to wheat and linseed matrices to release glucoside conjugates of 2,4-D, MCPA, dichlorprop, and haloxyfop. It should be noted that only glucoside conjugates were studied, and no ester derivatives of these herbicides were included in the evaluation. Samples were incubated for 24 h at 37 °C in acetate buffer (pH 4.0), then extracted with acidified acetonitrile and analyzed by LC–MS/MS. The study demonstrated that enzymatic hydrolysis provided satisfactory apparent recoveries across the tested fortification levels (10–50 µg kg^−1^). For 2,4-D glucoside, recoveries ranged from 95 to 107% in wheat and 99–109% in linseed, while dichlorprop glucoside recoveries were 91–99% in wheat and 77–102% in linseed. MCPA glucoside exhibited slightly lower recoveries (67–79% in wheat and 68–88% in linseed), whereas haloxyfop glucoside yielded the highest recoveries, ranging from 106 to 115% in wheat and 89–104% in linseed. Relative standard deviations (RSDs) were generally below 12%, indicating acceptable method repeatability [30]. The recovery results obtained in the present study clearly highlight differences among the four extraction and hydrolysis strategies. Option 2, representing the enzymatic and acid-assisted QuEChERS approach, consistently provided the highest recoveries across most analytes, ranging from 68.7% for fluroxypyr to 105.5% for fluazifop. This method can therefore be considered the most effective and reliable for releasing the target acidic herbicides from the tested matrices. In comparison, Option 1, which employed enzymatic hydrolysis alone, resulted in significantly lower recoveries for many analytes, including fluroxypyr (39.2%), haloksyfop (35.1%), and 2,4-DB (59.25%) (Table 8). These data indicate that, under the applied conditions, enzymatic deconjugation was insufficient to fully release the conjugated forms of several herbicides. Option 3, based on the addition of 5 M H_2_SO_4_, yielded high recoveries for some analytes, such as 2,4,5-T (92.6%) and fluazifop (100.1%), but was less consistent overall, with lower recoveries for quizalofop-P (60.4%) and haloxyfop (96.4%). Option 4, which involved only water addition, gave the lowest recoveries in most cases, demonstrating that the absence of hydrolytic or acidifying agents limits the efficiency of analyte release (e.g., 2,4-D: 27.5%; haloxyfop: 46.8%). These findings indicate that, while enzymatic hydrolysis is selective and avoids potential degradation of labile compounds, acid-assisted extraction ensures more complete recovery of a broader range of acidic herbicides under the tested experimental conditions.

Koesukwiwat et al. (2018) developed and validated a method for determining total haloxyfop residues in infant food matrices [33]. The authors demonstrated that complete conversion of haloxyfop-methyl ester to the free acid form was achievable only in a methanolic environment, with precise pH adjustment to approximately 2.5 via sulfuric acid. The hydrolysis was performed at ambient temperature for 2 h, followed by acetonitrile extraction and clean-up using dispersive solid-phase extraction with 150 mg of magnesium sulfate (MgSO_4_) and 50 mg sodium chloride (NaCl) (4:1, *w*/*w*). Notably, it was critical to avoid the use of PSA sorbent, which retained haloxyfop due to its carboxylic group, and to introduce hexane during sample preparation of high-fat matrices to reduce matrix co-extractives. The method demonstrated high accuracy (mean recoveries between 92 and 114%) and precision (RSD ≤ 14%) across a range of complex matrices, including infant formula, maltodextrin, soy protein isolate, and lecithin [33].

This study presents a hydrolysis (acidic and alkaline) QuEChERS-based method optimized for LC-MS/MS detection of acidic pesticide residues across diverse food matrices. In contrast, Lehner et al. (2020) focused on determining haloxyfop in eggs using GC-MS/MS after derivatization [34]. Our method enables the detection of multiple analytes at LOQs ranging from 0.01 to 0.2 mg/kg (10–200 µg/kg). Alkaline hydrolysis at 60 °C for 1 h effectively deconjugates ester and acid-labile forms, with recovery rates of 89–112% and RSDs ≤ 12%. Acid hydrolysis is applied at 60 °C for 16 h, mainly for bound glycosidic conjugates in plant-derived matrices. Lehner’s method achieved an LOQ of 2.5 ng/g (2.5 µg/kg) for haloxyfop-TMS in eggs and detected levels ranging from 2.7 to 14.5 ng/g in five of six batches. Their hydrolysis step used room temperature conditions for 2 h and required BSTFA + 1% TMCS derivatization, increasing sample handling complexity [34].

A comparative evaluation of the three hydrolysis strategies investigated in this study—enzymatic, acidic, and alkaline—revealed clear differences in analyte recovery and analytical precision (Table 9 and Table 10). The enzymatic approach (Options 1–4) showed considerable variability between compounds and hydrolysis variants. Recovery rates obtained by enzymatic hydrolysis ranged from approximately 27.5% to 120.3%, depending on the analyte and enzyme composition. For several compounds, including 2,4-D and haloxyfop, the enzymatic approach yielded relatively low recoveries in some variants (below 30%), suggesting incomplete cleavage of conjugated residues or insufficient hydrolysis efficiency. Although certain analytes, such as fluazifop or 2,4-DB, showed acceptable recoveries under specific enzymatic conditions, the overall variability between enzyme combinations suggests limited robustness of this approach for routine multi-residue analysis.

In contrast, the chemical hydrolysis protocols demonstrated more consistent analytical performance. The acid hydrolysis approach provided recoveries generally within the acceptable range defined by current validation guidelines (typically 70–120%) for the majority of analytes. At the LOQ level, recoveries ranged from approximately 58.5% to 107.8%, with very good repeatability, as indicated by relative standard deviations typically below 5.2%. Similar recovery levels were observed at higher fortification levels (5 × LOQ and 10 × LOQ), confirming the stability and reproducibility of the acidic hydrolysis conditions.

The alkaline hydrolysis protocol also enabled the release of several acidic herbicides; however, a greater variability in recovery values was observed compared with the acidic approach. In particular, certain analytes such as quizalofop-P showed substantial signal enhancement, suggesting strong matrix-induced effects or possible analyte transformation under alkaline conditions. Additionally, several compounds (e.g., 2,4-DB and fluroxypyr) exhibited lower recoveries and higher RSDs than with the acid hydrolysis procedure.

Overall, the comparative results indicate that while enzymatic hydrolysis may release selected conjugated residues, its performance depends strongly on enzyme composition and activity, resulting in variable recoveries. Alkaline hydrolysis improves analyte release for some compounds. In contrast, acidic hydrolysis provided the most balanced analytical performance, combining acceptable recoveries with good repeatability across the investigated analytes and concentration levels.

The suitability of the proposed method for routine residue analysis was evaluated in the context of current high-throughput analytical workflows. QuEChERS-based methodologies are widely recognized as state-of-the-art approaches due to their efficiency, minimal solvent consumption, and scalability [35,36]. Although acidic hydrolysis requires extended reaction time, it does increase active processing time and provides superior compatibility with LC-MS/MS and GC-MS/MS systems by eliminating the need for post-extraction pH adjustment, thereby enhancing method robustness and reproducibility [37]. Furthermore, such streamlined workflows are increasingly emphasized in regulatory and high-throughput laboratory settings, where analytical reliability, cost-efficiency, and operational simplicity are critical [38].

### 4.2. Effect of Physicochemical Properties and Extraction Conditions on Analytical Performance

Most of the evaluated active substances in this study are highly polar, hydrophilic compounds, predominantly belonging to the group of phenoxy acids and aryloxycarboxylic acid derivatives. Their ionizable character, reflected by relatively low pKa values (typically below 5), necessitates careful selection of extraction solvents and pH conditions to maximize analyte recovery from plant matrices. At pH 4.5, most of the studied active substances exist predominantly in partially or fully ionized forms. In this work, acetonitrile acidified with 1% (*v*/*v*) acetic acid was used as the extraction medium, enabling high extraction efficiency for both broad bean (*Vicia faba*) and common bean (*Phaseolus vulgaris*) matrices. These matrices exhibit near-neutral pH; however, no statistically significant differences in recovery were observed due to the inherent pH of the matrices, confirming the robustness of the acidified protocol. These findings are consistent with Schusterová et al.’s (2019, 2025) reports, in which acetonitrile acidified with 1% formic acid was utilized for extraction [39,40]. Based on the findings, acidified acetonitrile (ACN) significantly improved the extraction efficiency, likely due to enhanced solubility and stabilization of polar analytes in the acidic environment. Acidified acetonitrile was a more effective extraction medium than non-acidified solvents, likely due to enhanced solvation and stabilization of polar analytes in an acidic environment. These findings align with previous studies, such as Schusterová et al. (2019, 2025), where acidified acetonitrile (1% formic acid) provided comparable extraction efficiencies for phenoxy herbicides, regardless of the acidifying agent used [39,40]. The analyte behaviour during LC-MS/MS analysis is strongly influenced by the degree of ionization under acidic conditions. The degree of ionization is directly related to the compound-specific acid dissociation constant (pKa), which determines the predominant ionic species at a given pH. At extraction and chromatographic pH near 4.5, compounds with pKa values below this threshold, such as 2,4-D (pKa 2.64), MCPA (3.07), bentazon (3.28), and haloxyfop (4.33), exist predominantly in their deprotonated (anionic) forms. These substances exhibit increased hydrophilicity and reduced retention on reversed-phase stationary phases (e.g., C18), while showing poor ionization efficiency in positive electrospray ionization (ESI^+^) mode due to the absence of protonatable groups under acidic conditions. However, their deprotonated form is advantageous for negative electrospray ionization (ESI^−^), in which such molecules are more readily ionized and produce intense [M − H]^−^ molecular ions. Consequently, at pH 4.5, the abovementioned compounds exhibit high detectability and robust signal responses in ESI^−^ mode. Compounds with pKa values closer to 4.5 (e.g., 2,4-DB, MCPB) may exist in a dynamic equilibrium between their ionized and unionized forms, leading to broader peaks, variable retention times, and fluctuations in ionization response. In such cases, matrix pH or uncontrolled fluctuations can significantly impact quantitative performance. This underlines the importance of precise pH control in both extraction and chromatographic conditions for the reliable quantification of these substances. In ESI^−^ mode, ionization efficiency is generally enhanced for deprotonated (anionic) species; however, for compounds near their pKa (e.g., 2,4-DB, MCPB), partial protonation at pH 4.5 may lead to suboptimal ionization due to the coexistence of neutral and ionic forms. Additionally, matrix effects and precise pH control during sample preparation can further influence ionization performance and analytical sensitivity.

Quizalofop-P and its ester derivatives, included in the residue definition, exhibited noticeable deviations in recovery compared with other analytes. In the present study, only quizalofop-P was experimentally evaluated, and its recoveries were moderate under acidic hydrolysis (55.6–58.5%) and higher under alkaline hydrolysis (73.3–86.6%), with relative standard deviations reaching up to 14.5% at the highest fortification level. These results indicate that alkaline hydrolysis enhances the release of quizalofop-related residues compared with acidic conditions, although increased variability was observed at elevated spiking levels. The relatively low recoveries in acidic hydrolysis may be due to incomplete cleavage of ester-type residues or to partial matrix interactions that affect analyte extraction efficiency. However, slightly elevated recoveries above 100% were observed for some other analytes, such as haloxyfop (up to 107.8%) and 2,4-DB (103.5%) under acidic hydrolysis, which may be attributed to minor matrix-induced signal enhancement during LC-MS/MS detection. The superior performance of acidic hydrolysis observed in this study is directly linked to the physicochemical properties of the target analytes. Most compounds possess low pKa values and exist predominantly in their anionic form at pH ~4.5, which enhances solubility in acidified acetonitrile and maximizes ionization efficiency in ESI^−^ mode. This translates into consistently high recoveries and good repeatability (RSDr values typically ranging from 0.7% to 5.8%) for the majority of compounds analysed using the acidified QuEChERS protocol. In contrast, alkaline hydrolysis—while improving recovery for some esterified residues—showed higher variability for several analytes, including 2,4-DB (RSDr up to 15.3%) and quizalofop-P (RSDr up to 14.5%), as well as lower recoveries for compounds such as fluroxypyr (45.6–59.3%). These results suggest that strong alkaline conditions may promote co-extraction of interfering matrix components, leading to increased variability in signal response during LC-MS/MS analysis.

In the study by Cheng et al., temperature has been identified as a critical factor influencing the degradation dynamics of propaquizafop in environmental matrices. In soil systems, elevated temperatures significantly accelerate both microbial and abiotic degradation pathways, leading to markedly reduced half-lives (e.g., from 7.75 to 0.54 days in unsterilised soil at 25–50 °C), as demonstrated in ginseng cultivation environments [41]. These findings underscore the analytical complexity of compounds subject to composite residue definitions and highlight the need for rigorously optimized hydrolytic protocols to ensure accurate quantification in line with SANTE/11312/2021 v2026 criteria [7].

The superior performance of acidic hydrolysis observed in this study is directly linked to the physicochemical properties of the target analytes. Most compounds possess low pKa values and exist predominantly in their anionic form at pH ~ 4.5, which enhances solubility in acidified acetonitrile and maximizes ionization efficiency in ESI^−^ mode. This translates into consistently high recoveries, excellent repeatability (RSDs < 3.5%), In contrast, alkaline hydrolysis—while improving recovery for some esterified residues—showed higher variability, broader peaks, and signs of matrix interferences. Therefore, acidic hydrolysis offers the best balance between extraction efficiency, precision, and signal clarity, making it the most suitable and reliable approach for routine analysis of acidic pesticides in complex food matrices.

### 4.3. Molecular Insights into Acid vs. Alkaline Hydrolysis in Pesticide Extraction

**Figure 1:**
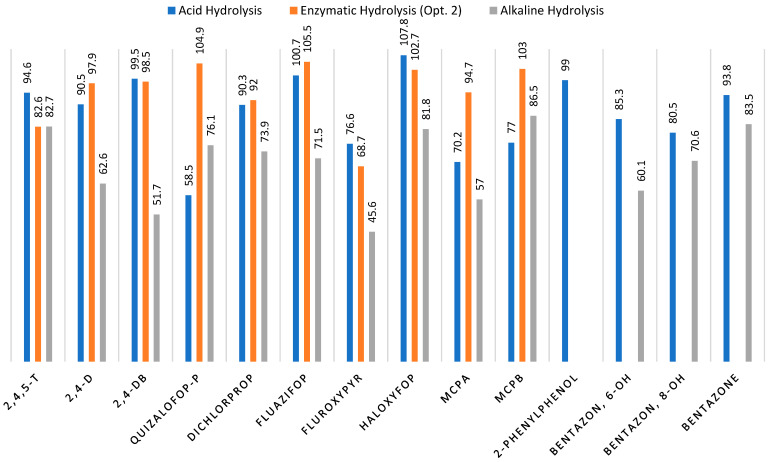
Comparison of the mean recovery of the acidic, alkaline, and enzymatic hydrolysis.

While acidic hydrolysis QuEChERS provides excellent results for free forms of acidic herbicides, bound (e.g., conjugated) residues often require hydrolysis for quantitative release. In this context, alkaline hydrolysis is a powerful tool for liberating ester- or glucoside-bound pesticide residues. The hydrolysis time differed due to the reaction mechanisms of the two processes. Acid hydrolysis was performed for 16 h to ensure complete cleavage of glycosidic and other bound conjugates present in plant matrices. In contrast, alkaline hydrolysis was performed for 1 h, as esterified residues undergo rapid, irreversible cleavage under basic conditions. Preliminary experiments confirmed that extending alkaline hydrolysis time did not statistically significantly affect analyte recoveries. The proposed alkaline QuEChERS protocol was based on pH adjustment to >12, which enables irreversible base-catalysed hydrolysis of conjugated moieties. Compared to acid hydrolysis, where reaction efficiency is strongly time-dependent and reversible, alkaline hydrolysis offers faster and more complete degradation of esters. Notably, compounds like quizalofop-ethyl hydrolyse more rapidly under basic conditions, forming the active acid, quizalofop. This behaviour is supported by the hydrolysis kinetics, which show maximal efficiency at extreme pH values (<1 or >12), with temperature acting as a critical accelerator of the reaction rate [42]. Nevertheless, the hydrolysis temperature must be optimized to prevent analyte degradation or adverse matrix transformations. Structurally, all tested analytes except bentazon and its hydroxylated derivatives are organic acids with hydrolysable ester bonds or conjugated forms. The hydrolysis of esters involves a well-established mechanism: under acidic conditions, protonation of the carbonyl oxygen increases electrophilicity, promoting nucleophilic attack by water, followed by rearrangement and elimination of the alcohol leaving group. In contrast, base-catalyzed hydrolysis involves direct nucleophilic attack by hydroxide anions, resulting in the formation of carboxylate salts and alcohols. This reaction is irreversible and independent of the presence of a catalyst, making it more efficient for robust sample preparation. The hydrolysis reactions are visualized in Figure 1, where both acid- and base-catalyzed pathways are schematically represented with resonance structures, emphasizing the role of carbonyl carbon reactivity, mesomeric stabilization, and inductive effects from alkyl substituents. However, high-pH extraction introduces additional complexity. Under strong alkaline conditions, co-extraction of interfering matrix components often intensifies, resulting in pronounced matrix effects. These effects can distort ionization efficiency and compromise analytical accuracy. Therefore, the application of appropriate purification steps using salt buffers (e.g., citrate or phosphate buffers) and dispersive solid-phase extraction (d-SPE) is essential to mitigate these issues. Purification is particularly critical for high-starch or high-protein matrices, such as legumes, where matrix interferences can significantly suppress or enhance signal response in MS detection. In light of this, despite the advantages of alkaline hydrolysis for analyte release and signal intensification, the overall method must balance hydrolysis efficiency with matrix cleanliness to ensure robust, reproducible analytical performance.

While alkaline hydrolysis has demonstrated high efficiency in releasing ester- and glucuronide-bound pesticide residues, acid hydrolysis remains a compelling alternative in analytical workflows due to several operational and analytical advantages. Notably, acid hydrolysis does not require extreme pH adjustment beyond the analytical range of chromatographic columns. This feature simplifies downstream analysis, as pH normalization can be effectively achieved using primary-secondary amine (PSA) sorbents without compromising column integrity or analyte stability. During method optimization, attempts to introduce a basic neutralization step resulted in precipitate formation and significantly increased relative standard deviations (RSD), underscoring the operational robustness of the acid-mediated approach. Moreover, acid hydrolysis offers improved compatibility with dispersive solid-phase extraction (d-SPE) and minimizes co-extraction of interfering matrix components, which are frequently observed under high-pH alkaline conditions. This translates into reduced matrix effects and more consistent ionization efficiency in mass spectrometric detection, particularly critical when analysing complex food matrices rich in proteins or starch. The milder conditions of acid hydrolysis also reduce the risk of analyte degradation and undesirable matrix transformations, thereby preserving the integrity of both target compounds and the sample composition. Collectively, these advantages render acid hydrolysis a practical and reliable strategy for the quantitative release of conjugated residues, particularly when matrix cleanliness and analytical reproducibility are of primary concern.

**Figure 2:**
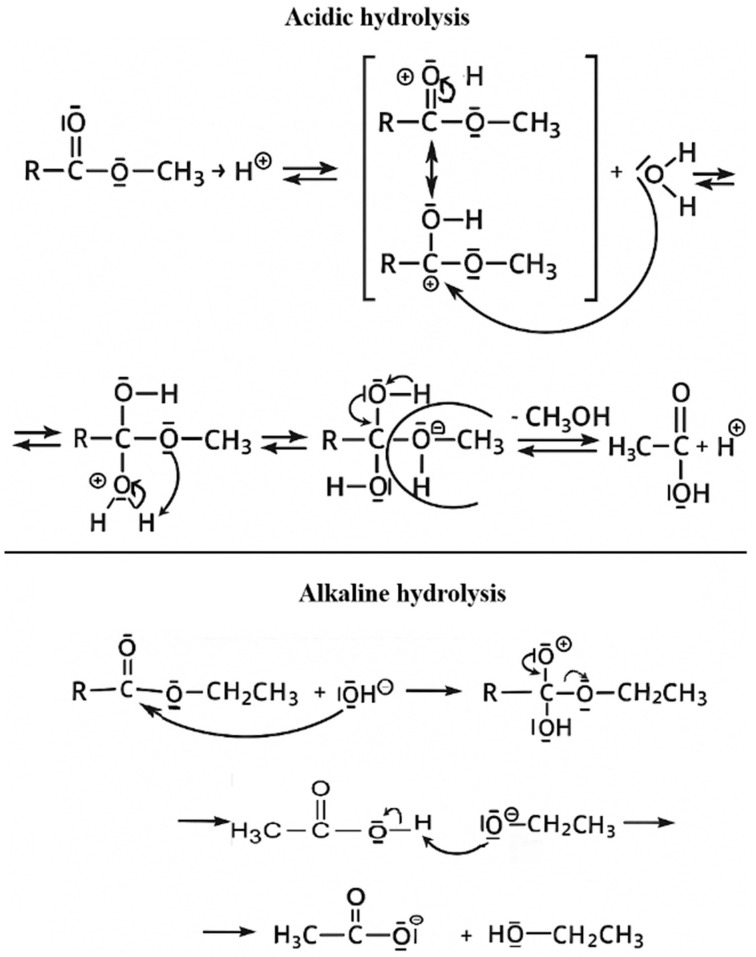
General molecular mechanism of acid- and alkaline-catalyzed hydrolysis of esters representing the basic chemical structure of pesticide residues.

The kinetics of the hydrolysis reaction indicate that the process is efficient at both acidic pH < 1 and basic pH > 12. The effect of temperature on the hydrolysis reaction rate is significant, substantially increasing the reaction rate; however, the temperature must be optimized to avoid the degradation of pesticides and matrix components. All analysed compounds except bentazone and its derivatives have the structure of organic acids. The hydrolysis mechanism involves two distinct pathways and involves various processes. Three atoms (two oxygen atoms and one carbon atom) are involved in the resonance stabilization of esters. Molecular formulas can be represented as appropriate mesmeric structures. A stabilizing factor in the system is the inductive effect of the alkyl group attached to the oxygen atom. For this reason, ester compounds are low-reactive and require stringent conditions for hydrolysis. In an acidic environment, hydrolysis is catalysed by mineral acid. Protonation of the carbonyl oxygen atom enhances the electrophilic interaction of the carbon (Csp2) with the nucleophile H_2_O. In the second stage of the reaction, after an intramolecular rearrangement, the alcohol molecule (the leaving group) is eliminated. Acidic hydrolysis of esters constitutes a nucleophilic substitution reaction in the acyl group. The ester hydrolysis mechanism in an aqueous environment is irreversible. As a result, an alcohol and a carboxylate salt are formed. In alkaline hydrolysis, OH^−^ ions act as reactants rather than catalysts. Both hydrolysis reaction mechanisms are illustrated in Figure 2.

## 5. Conclusions

This study demonstrates that the reliability of acidic pesticide residue determination in complex legume matrices is strongly dependent on the selected hydrolysis strategy and its interaction with analyte physicochemical properties. The experimental data show that acidified QuEChERS extraction provides the most consistent analytical performance, combining moderate-to-high recoveries (55.6–107.8%) with low variability (RSD typically <5%) and stable chromatographic behavior. Notably, recoveries at the lower end of this range should be interpreted as moderate rather than high and are consistent with literature reports for polar, matrix-sensitive analytes in complex plant systems. Alkaline hydrolysis improved the release of selected esterified residues, as reflected by increased recoveries for compounds such as quizalofop-P; however, this was accompanied by significantly higher variability (RSD up to 14.5%) and enhanced matrix effects, indicating reduced method robustness. These findings are consistent with previous reports highlighting the trade-off between hydrolysis efficiency and matrix co-extraction under strongly basic conditions. The ionization behavior of the analytes was governed by their physicochemical properties, particularly their pKa values, which influenced both extraction efficiency and LC-MS/MS signal response. Compounds predominantly present in anionic form at pH 4.5 showed stable responses and reproducible chromatographic performance, whereas analytes near their dissociation equilibrium exhibited greater variability, underscoring the importance of strict pH control. Compared to GC-MS/MS methods requiring derivatization for compounds such as haloxyfop, the applied LC-ESI-MS/MS approach enables direct determination, reducing sample preparation complexity and potential sources of analytical variability. Although both protocols fulfilled the SANTE/11312/2021 v2026 validation criteria [7], the results indicate that method performance remains analyte- and matrix-dependent. Overall, acidified extraction represents the most robust general approach for routine multi-residue analysis, while alkaline hydrolysis should be applied selectively when enhanced release of specific conjugated residues is required.

## Figures and Tables

**Table 1:** Pesticide Residue Exceedances in Food Crops (EFSA, 2022–2023).

Pesticide	2022			2023		
	Total Results	Above the Legal Limit	%	Total Results	Above the Legal Limit	%
2,4,5-T	12.040	0	0.00%	6892	0	0.00%
2,4,5-TP	12.393	0	0.00%	12,526	0	0.00%
2,4-D	22.208	6	0.03%	21,665	11	0.05%
2,4-DB	15.446	0	0.00%	14,600	0	0.00%
2-Phenylphenol	37.599	15	0.04%	41,696	12	0.03%
Dichlorprop	15.324	1	0.01%	16,488	2	0.01%
MCPA and MCPB	22.390	4	0.02%	23,087	2	0.01%
Haloxyfop	27.157	55	0.21%	27,146	29	0.11%
Fluazifop	31.422	23	0.07%	34,282	10	0.03%
Propaquizafop	36.659	0	0.00%	42,605	0	0.00%
Quizalofop	18.126	2	0.04%	20,425	1	0.00%
Fluroxypyr	20.014	0	0.00%	10,527	2	0.02%
Bentazone	18.313	0	0.00%	23,290	0	0.00%

**Table 2:** Composition of the spiking mixture containing the parent compounds and their determined concentrations (ng/mL).

Name Compound	Concentration in Definition (ng/mL)
2,4-D	50
2,4-DB	50
2,4,5-T acid	10
2-phenylophenol	10
Dichlorprop	20
MCPA	25
MCPB	25
Haloxyfop	10
Fluazifop	10
Quizalofop-P	100
Propaquzalfop	100
Fluroxypyr	10
Bentazone	16.67
Bentazone-6-hydroxy	16.67
Bentazone-8-hydroxy	16.67

**Table 3:** Composition of the spiking mixture containing individual esters, conjugates, and metabolites included in the complex residue definitions for selected pesticides. For each pesticide, the corresponding molecular weight (MW) and conversion factor (CF) are listed for the individual analytes defined within the respective residue definition.

Definition	Name Compound	MW	Conversion Factor (CF)	Concentration in Definition (ng/mL)	Concentration of Compounds (ng/mL)
2,4-D (sum of 2,4-D, its salts, its esters and its conjugates, expressed as 2,4-D)	2,4-D	221.0	1.00	50	8.33
	2,4-D-Methylester	235.1	0.94		8.33
	2,4-D-1-buthyl ester	277.1	0.80		8.33
	2,4-D isoprophyl ester	263.1	0.84		8.33
	2,4-D-iso-buthyl ester	277.1	0.80		8.33
	2,4-D 2-ethylhexyl ester	333.3	0.66		8.33
2,4-DB (sum of 2,4-DB, its salts, its esters and its conjugates, expressed as 2,4-DB)	2,4-DB	249.1	1.00	50	25.00
	2,4-DB methyl ester	263.1	0.89		25.00
2,4,5-T (sum of 2,4,5-T, its salts and esters, expressed as 2,4,5-T)	2,4,5-T acid	255.5	1.00	10	3.33
	2,4,5-T n-butyl ester	311.6	0.75		3.33
	2,4,5-T-methyl ester	269.5	0.87		3.33
2-phenylphenol (sum of 2-phenylphenol and its conjugates, expressed as 2-phenylphenol)	2-phenylophenol	170.21	1.00	10	5.00
	2-fphenylophenol-glucoside	332.35	0.51		5.00
Dichlorprop (Sum of dichlorprop (including dichlorprop-P), its salts, esters and conjugates, expressed as dichlorprop)	Dichlorprop	235.1	1.00	20	5.00
	Dichlorprop-p	235.1	1.00		5.00
	Dichlorprop-methyl ester	249.1	0.94		5.00
	Dichlorprop-2-ethylhexyl	347.3	0.68		5.00
MCPA and MCPB (MCPA, MCPB including their salts, esters and conjugates expressed as MCPA)	MCPA	200.6	1.00	25	4.17
	MCPA-2-ethylhexyl ester	312.8	0.64		4.17
	MCPA methyl ester	214.6	0.93		4.17
	MCPA-ethyl ester	228.7	0.88		4.17
	MCPA-1-buthyl ester	256.7	0.78		4.17
	MCPA-thioethyl	244.7	0.82		4.17
	MCPB	228.7	0.88	25	12.50
	MCPB-ethyl	256.7	0.89		12.50
Haloxyfop (Sum of haloxyfop, its esters, salts and conjugates expressed as haloxyfop (sum of the R- and S- isomers at any ratio))	Haloxyfop	361.7	1.00	10	3.33
	Haloxyfop-methyl	375.7	0.96		3.33
	Haloxyfop-2-ethoxyethyl	433.8	0.83		3.33
Fluazifop-P (sum of all the constituent isomers of fluazifop, its esters and its conjugates, expressed as fluazifop)	Fluazifop	327.3	1.00	10	2.50
	Fluazifop-P	327.3	1.00		2.50
	Fluazifop-methyl	341.3	0.96		2.50
	Fluazifop-P-buthyl	383.4	0.85		2.50
Quizalofop (sum of quizalofop, its salts, its esters (including propaquizafop) and its conjugates, expressed as quizalofop (any ratio of constituent isomers))	Quizalofop-P	344.7	1.00	200	50.00
	Propaquzalfop	443.9	0.57		50.00
	Quizalofop-ethyl	372.8	0.68		50.00
	Quizalofop methyl	358.8	0.96		50.00
Fluroxypyr (sum of fluroxypyr, its salts, its esters, and its conjugates, expressed as fluroxypyr)	Fluroxypyr	255.0	1.00	10	5.00
	Fluroxypyr-1-methylheptyl ester	367.2	0.69		5.00
Bentazone (Sum of bentazone, its salts and 6-hydroxy (free and conjugated) and 8-hydroxy bentazone (free and conjugated), expressed as bentazone)	Bentazone	240.1	1.00	50	16.67
	Bentazone-6-hydroxy	256.1	1.00		16.67
	Bentazone-8-hydroxy	256.1	1.00		16.67

**Table 4:** Schematic overview of the enzymatic and chemical hydrolysis variants applied prior to QuEChERS extraction and LC–MS/MS analysis.

Stage	Option 1	Option 2	Option 3	Option 4
Buffer	Adding 10 mL buffer acetate pH 4.			
Fortification	Adding standards to spiked samples			
Enzyme	β-glucosidase 5 U/mL β-galactosidase 5 U/mL α-amylase 5 U/mL		Not added	Not added
Conditions for hydrolysis	Incubation for 4 h at 37 °C in a water bath with shaking			
	10 mL 1% acetic acid in ACN			
	+2 mL H_2_O	+2 mL 5M H_2_SO_4_	+2 mL 5M H_2_SO_4_	+2 mL H_2_O
	Incubation for 16 h at 60 °C in a water bath with shaking			
Extraction QuEChERS	Add QuEChERS salt (4 g MgSO_4_, 1 g NaCl, 1 g TSCD, 0.5 g DHS)			
	Cool in an ice bath for 5 min, shake for 2 min, centrifuge for 5 min, at 10 °C at 4000 rpm.			
	6 mL of extract was taken, and salts were added (900 mg MgSO_4_, 150 mg PSA).			
	Cool in an ice bath for 5 min, shake for 2 min, centrifuge for 5 min at 10 °C at 4000 rpm.			
	Filtering the extract through a 0.22 µm PTFE filter.			
Method analysis	LC-MS/MS			

**Table 5:** LC-MS/MS conditions and target pesticides. For each compound, the retention time (tR), ion polarity, precursor and product ion transitions, fragmentor voltage, cell acceleration voltage (CAV), and collision energy (CE) are provided.

LC-MS/MS Conditions for the Target Pesticides and the Derivative Compounds						
Target Analyte	RT (min)	Polarity	SRMs	Fragmentor	CAV (V)	CE (eV)
			(*m*/*z*)	(V)		
2,4,5-T	21	Negative	254.9 → 196.9	80	3	10
			252.9 → 194.9	80	3	10
			252.9 → 158.9	80	3	40
			252.9 → 122.9	80	3	45
			252.9 → 95.0	80	3	60
2.4-D	19	Negative	221.0 → 162.9	64	4	12
			219.0 → 160.9	80	4	13
			219.0 → 160.9	88	4	16
2.4-DB	22	Negative	249.0 → 163.0	80	3	10
			247.0 → 161.0	80	3	10
Quizalofop-P	23	Negative	343.0 → 271.1	86	2	12
			343.0 → 243.1	86	2	28
			343.0 → 108.0	86	2	48
			343.0 → 35.1	86	2	44
Dichlorprop	21	Negative	335.0 → 163.0	80	4	10
			233.0 → 161.0	80	4	10
Fluazifop	21	Positive	328.0 → 282.1	145	4	16
			328.0 → 254.1	145	3	24
			328.0 → 238.1	145	4	32
			328.0 → 233.1	145	3	36
			328.0 → 146.1	145	4	56
Fluoxypyr	17	Negative	353.0 → 232.9	71	2	1
			353.0 → 194.9	71	2	13
Haloxyfop	23	Positive	362.1 → 316.2	120	4	12
			362.1 → 288.1	120	4	24
			362.1 → 91.0	120	4	28
			362.1 → 65.1	120	4	60
MCPA	19.1	Negative	201.0 → 143.0	100	4	15
			199.0 → 141.0	100	4	15
MCPB	21	Negative	229.1 → 143.0	80	4	5
			229.1 → 141.0	80	4	5
Bentazone-6-hydroxy	12.5	Negative	255.0 → 191.0	113	2	16
			255.0 → 147.9	113	2	28
Bentazone-8-hydroxy	12.5	Negative	255.0 → 191.0	121	2	16
			255.0 → 148.0	121	2	24
			255.0 → 108.0	121	2	28
			255.0 → 106.1	121	2	28
Bentazone	13.5	Negative	239.0 → 197.0	118	4	16
			239.0 → 175.0	118	4	16
			239.0 → 132.0	118	4	24
Propaquizalofop	22.2	Positive	444.1 → 370.0	125	2	12
			444.1 → 299.2	125	2	20
			444.1 → 100.1	125	2	16

**Table 6:** GC-MS/MS conditions and target pesticides. Name of compound, the retention time (tR), Index Retention Kovats in HP-5ms, precursor and product ion transitions, and collision energy (CE) are provided.

GC-MS/MS Conditions for the Target Pesticides and the Derivative Compounds				
Target Analyte	RT (min)	IR Kovats	SRMs	CE (eV)
			(*m*/*z*)	
2-phenylophenol	11.1	1513	170.1 → 141.1	24
			141.1 → 115.1	18
			170.1 → 115.1	28

**Table 7:** Matrix effect of alkaline and acid hydrolysis.

Name Compounds	Alkaline Hydrolysis	Acid Hydrolysis
	[%]	[%]
2,4,5-T	33.9	43.0
2,4D	60.3	68.2
2,4-DB	29.5	29.0
Bentazon, 6-hydroksy	35.5	29.7
Bentazon, 8-hydroksy	83.1	85.1
Bentazone	31.7	29.1
Chizalofop-P	32.8	28.1
Dichlorprop	131.0	135.5
Fluazifop	29.6	34.2
Fluroksypyr	27.2	0.2
Haloksyfop	6.8	71.9
MCPA	75.6	88.2
MCPB	18.7	27.6
Propaquizafop	18.1	52.7
2-phenylphenol	-	56.3

**Table 8:** Pesticide residue analysis results obtained by enzymatic hydrolysis. The corresponding spiking level and recovery value (REC [%]) for each pesticide are presented.

Analyte	1·LOQ	Option 1 REC [%]	Option 2 REC [%]	Option 3 REC [%]	Option 4 REC [%]
	[mg·kg^−1^]				
2,4,5-T	0.010	64.1	82.6	92.6	65.8
2,4 D	0.050	69.7	97.9	73.9	27.5
2,4-DB	0.050	59.25	98.5	108.9	64.1
Chizalofop-P	0.100	84.9	104.9	60.4	40.3
Dichlorprop	0.020	62.0	92.0	92.5	76.6
Fluazifop	0.010	79.1	105.5	100.1	78.9
Fluroksypyr	0.010	39.2	68.7	71.0	40.8
Haloksyfop	0.010	35.1	102.7	96.4	46.8
MCPA	0.025	79.3	94.7	98.7	80.7
MCPB	0.025	78.0	103.0	89.0	87.0

**Table 9:** Pesticide residue analysis results obtained by the acid hydrolysis. The corresponding spiking level, recovery value (REC [%]), and relative standard deviation (RSDr [%]) for each pesticide are presented.

	Acidified QuEChERS									
Analyte	MRL	1·LOQ	REC [%]	RSDr [%]	5·LOQ	REC [%]	RSDr [%]	10·LOQ	REC [%]	RSDr [%]
	[mg·kg^−1^]	[mg·kg^−1^]			[mg·kg^−1^]			[mg·kg^−1^]		
2,4,5-T	0.01	0.010	94.6	2.7	0.050	95.2	3.9	0.10	94.6	2.7
2,4 D	0.01	0.050	90.5	2.2	0.25	90.1	0.9	0.50	90.5	2.2
2,4-DB	0.01	0.050	99.5	1.7	0.25	103.5	2.8	0.50	99.5	1.7
2-phenylphenol	0.01	0.010	99.0	2.0	0.050	101.0	1.0	0.10	98.0	2.0
Bentazon, 6-hydroxy	0.05	0.017	85.3	2.7	0.083	86.5	2.0	0.17	85.3	2.7
Bentazon, 8-hydroxy		0.017	80.5	2.0	0.083	78.0	1.3	0.17	80.5	2.0
Bentazone		0.017	93.8	2.3	0.083	94.5	1.7	0.17	93.8	2.3
Quizalofop-P	0.2	0.100	58.5	3.6	0.50	55.6	2.6	1.0	58.5	3.6
Dichlorprop	0.02	0.020	90.3	4.6	0.10	92.9	1.8	0.20	90.3	4.6
Fluazifop	0.01	0.010	100.7	5.2	0.050	104.7	5.8	0.10	100.7	5.2
Fluroxypyr	0.01	0.010	76.6	4.9	0.050	71.3	2.0	0.10	76.6	4.9
Haloxyfop	0.01	0.010	107.8	5.2	0.050	97.4	5.7	0.10	107.8	5.2
MCPA	0.1	0.025	70.2	1.2	0.13	70.2	0.7	0.25	70.2	1.2
MCPB		0.025	77.0	3.0	0.13	82.2	2.4	0.25	77.0	3.0

**Table 10:** Pesticide residue analysis results for the alkaline hydrolysis method. For each pesticide, the corresponding spiking level, recovery value (REC [%]), and relative standard deviation (RSDr [%]) are presented.

Alkaline QuEChERS										
Analyte	MRL	1·LOQ	REC [%]	RSDr [%]	5·LOQ	REC [%]	RSDr [%]	10·LOQ	REC [%]	RSDr [%]
	[mg·kg^−1^]	[mg·kg^−1^]			[mg·kg^−1^]			[mg·kg^−1^]		
2,4,5-T	0.01	0.010	82.7	6.9	0.050	83.6	4.0	0.10	95.3	7.9
2,4 D	0.01	0.050	62.6	1.2	0.25	74.6	4.1	0.50	77.3	9.3
2,4-DB	0.01	0.050	51.7	15.3	0.25	61.5	3.7	0.50	62.8	1.9
Bentazon, 6-hydroxy	0.05	0.017	60.1	8.2	0.083	83.0	7.5	0.17	96.6	3.9
Bentazon, 8-hydroxy		0.017	70.6	1.4	0.083	89.2	6.8	0.17	95.7	2.0
Bentazone		0.017	83.5	2.1	0.083	96.7	3.3	0.17	100.8	1.1
Quizalofop-P	0.2	0.100	76.1	6.7	0.50	86.6	4.3	1.0	73.3	14.5
Dichlorprop	0.02	0.020	73.9	5.2	0.10	86.3	3.3	0.20	86.7	8.9
Fluazifop	0.01	0.010	71.5	2.9	0.050	80.5	6.0	0.10	82.8	12.5
Fluroxypyr	0.01	0.010	45.6	10.1	0.050	59.3	7.7	0.10	51.9	8.6
Haloxyfop	0.01	0.010	81.8	11.7	0.050	91.8	3.5	0.10	86.3	18.2
MCPA	0.1	0.025	57.0	5.2	0.13	67.5	4.7	0.25	71.9	8.7
MCPB		0.025	86.5	3.2	0.13	88.3	3.2	0.25	88.3	1.6

## Data Availability

Data supporting the reported results are stored at the Voivodship Sanitary and Epidemiological Station in Warsaw.
